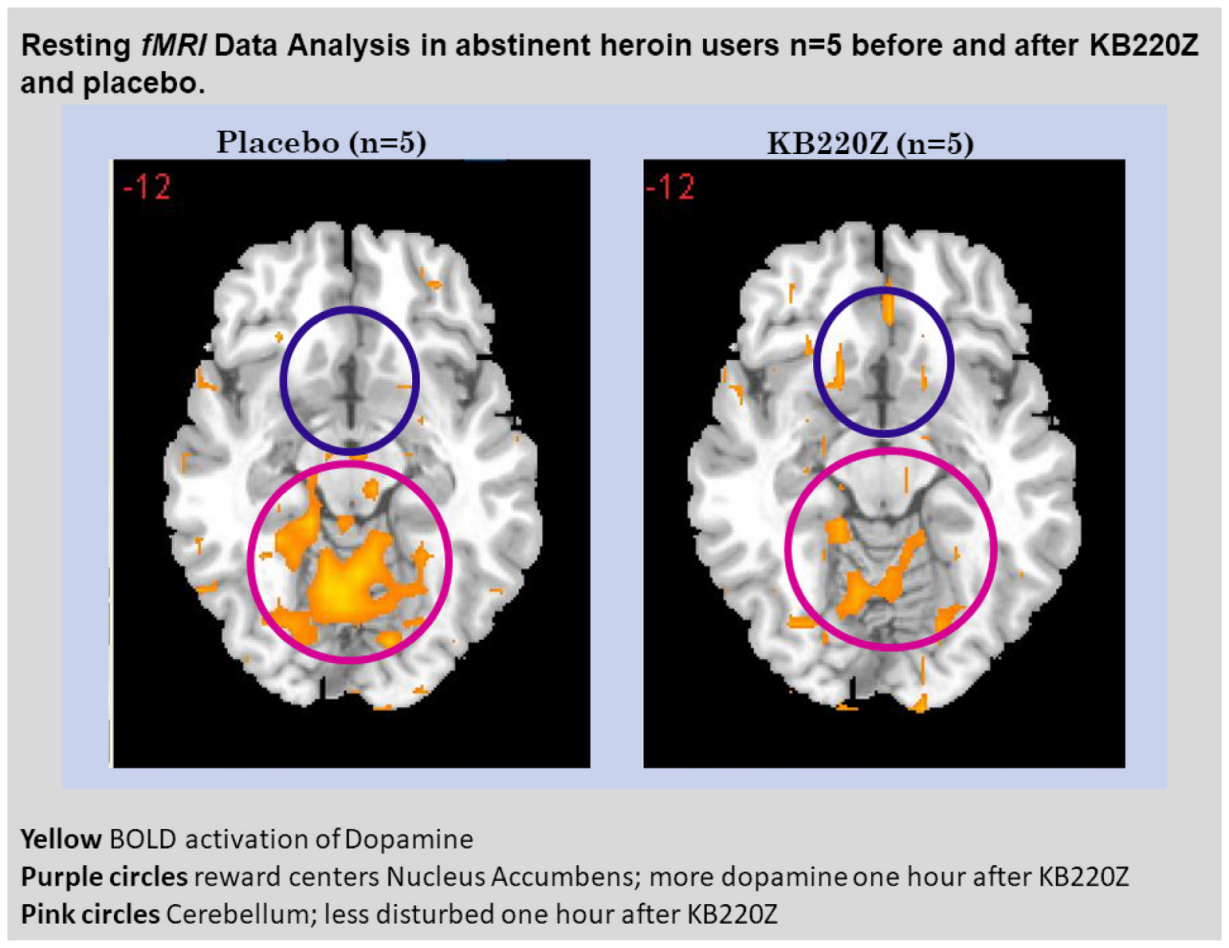# “Dopamine homeostasis” requires balanced polypharmacy: Issue with destructive, powerful dopamine agents to combat America’s drug epidemic

**DOI:** 10.15761/JSIN.1000183

**Published:** 2017-11-17

**Authors:** Kenneth Blum, Edward J Modestino, Marjorie Gondré-Lewis, B William Downs, David Baron, Bruce Steinberg, David Siwicki, John Giordano, Thomas McLaughlin, Jennifer Neary, Mary Hauser, Lyle Fried, Rajendra D Badgaiyan

**Affiliations:** 1 Department of Psychiatry, University of Florida & McKnight Brain Institute, College of Florida, Gainesville, FL, USA; 2 Department of Psychiatry, Human Integrated Services Unit University of Vermont Center for Clinical & Translational Science, College of Medicine, Burlington, VT, USA; 3 Dominion Diagnostics, LLC, North Kingstown, RI, USA; 4 Department of Psychiatry, Wright State University, Boonshoft School of Medicine, Dayton, OH USA; 5 Division of Genetic Testing, Geneus Health LLC, San Antonio, Texas, USA; 6 Center for Genomics and Applied Gene Technology, Institute of Integrative Omics and Applied Biotechnology (IIOAB), Nonakuri, Purba Medinipur, West Bengal, India; 7 Institute of Psychology, Eötvös Loránd University Budapest, Hungary; 8 Department of Psychiatry, University of Southern California, Keck School of Medicine, Los Angeles, CA, USA; 9 Division of Addiction Research & Therapy, The Shores Treatment & Recovery Center, Port St Lucie, Fl, USA; 10 Department of Psychology, Curry College, Milton, MA, USA; 11 Departments of Anatomy and Psychiatry and Behavioral Sciences, Howard University, Washington, DC, USA; 12 Victory Nutition International, Inc., Lederach, PA, USA; 13 John Giordano’s Life Enhancement Aftercare Recovery Center, Ft. Lauderdale, FL, USA; 14 Center for Psychiatric Medicine, North Andover, MA, USA; 15 Richmond University Hospital, Ichan Medical School, Statin Island, NY, USA

**Keywords:** D2 agonists, dopamine antagonists, dopamine homeostasis, drug epidemic, KB220, KB220Z

## Abstract

The well-researched pro-dopamine regulator KB220 and variants result in increased functional connectivity in both animal and human brains, and prolonged neuroplasticity (brain cell repair) having been observed in rodents. Moreover, in addition to increased functional connectivity, recent studies show that KB220Z increases overall brain connectivity volume, enhances neuronal dopamine firing, and eliminates lucid dreams in humans over a prolonged period. An unprecedented number of clinical studies validating this patented nutrigenomic technology in re-balancing brain chemistry and optimizing dopamine sensitivity and function have been published. On another note, it is sad that unsuspecting consumers could be deceived and endangered by false promises of knock-off marketers with look- and- sound-alike products. Products containing ingredients having potential dangers (i.e., combinations of potent D2 agonists including L-Dopa and L-Theanine) threaten the credibility and reputation of validated, authentic, and ethical products. We encourage clinicians and neuroscientists to continue to embrace the concept of “dopamine homeostasis” and search for safe, effective, validated and authentic means to achieve a lifetime of recovery, instead of reverting to anti-dopaminergic agents doomed to fail in the war against the devastating drug epidemic, or promoting powerful D2 agonists that compromise needed balance.

## Introduction

In 1908, President Theodore (Teddy) Roosevelt, worried that the national crisis of opiate addiction was weakening America and diminishing its greatness. Therefore, he appointed an Ohio doctor, Hamilton Wright, to be the nation’s first Opium Commissioner. In the decades after the Civil War, the United States developed a deadly narcotics habit. Suffering veterans were “hooked on morphine,” while genteel “society ladies” dosed up with laudanum—a tincture of alcohol and opium. The wonder drug was used widely as a cough suppressant, and proved very effective in treating diarrhea in children. In fact, in 1911, Wright told the NY Times —*”Our prisons and our hospitals are full of victims of it; it has robbed ten thousand businessmen of moral sense and made them beasts who prey upon their fellows it has become one of the most fertile causes of unhappiness and sin in the United States”*

Remarkably, more than a century later, America has relapsed [1]. The current opioid crisis is more lethal, with record numbers of fatal overdoses, public health professionals expose report. However, it is not the first time in U.S. history that the lax commercialization of legal opioids led to a national epidemic. To reiterate, faced with a late 19th-century dope scourge, federal law enforcement officials, doctors, and pharmacists eventually managed to contain the country’s first addiction epidemic [2].

**Figure 1:**
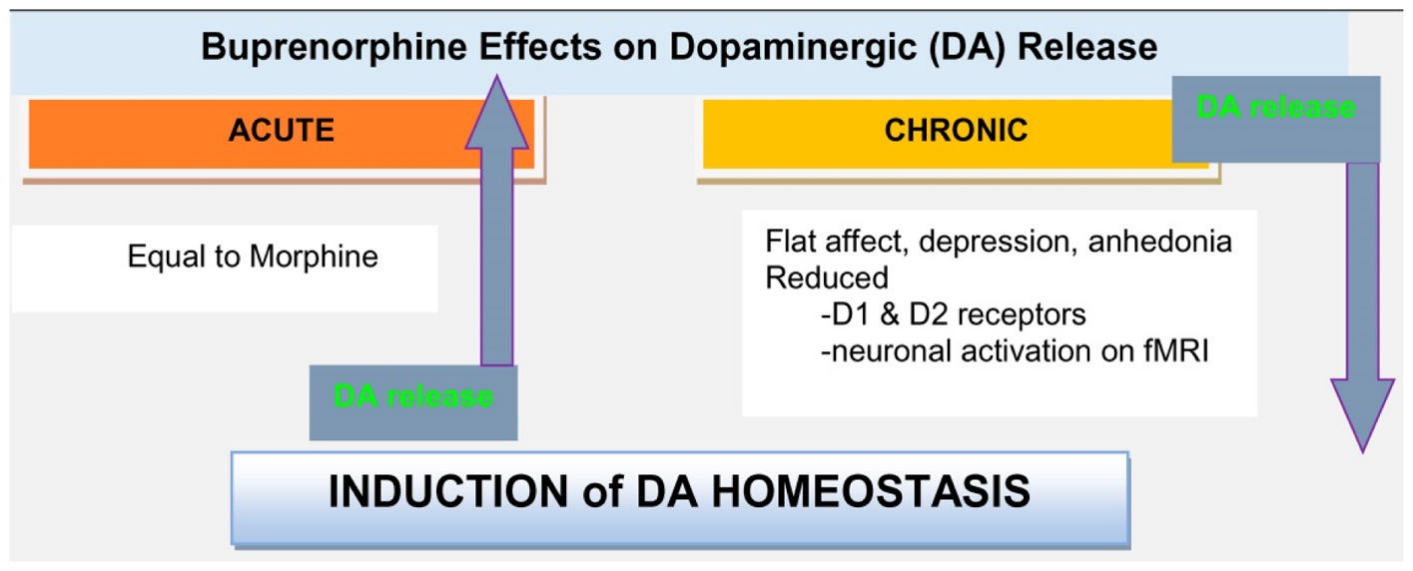
A graphical abstract showing short- and long-term administration of buprenorphine on dopamine release at the brain reward site (nucleus accumbens) [5].

The authors, who have numerous scientific publications on this issue, believe that FDA approved Medication Assisted Treatments (MATS), such as, the acute administration of buprenorphine, are helpful in inducing short-term dopamine release [3–5]. Chronic use, however, induces a significant reduction in dopamine release at the reward site of the brain, causing an unwanted, anti-reward state (see Figure 1) [4]. It is not yet definativly known what the exact biological consequences of chronic exposure to partial agonist or full antagonist, such as, in the form of buprenorphine or naltrexone, respectively, will be on brain function. It is important to consider that the current therapies require chronic receptor activity or blockades and are not natural means of restoring dopamine homeostasis.

For more than 50 years, our research has provided a significant dossier of peer-reviewed and published evidence in scientific journals showing that balancing dopamine dynamics in the brain reward circuitry is a far more desirable and useful strategy than blocking its *normal* physiologically required function [6]. Fundamentally, dogmatic protocols, such as methadone treatment, used routinely in addiction treatment are an attempt to medicate people with substance dependence back to health. To put it more simply, we are trying to force health rather than nourish the body’s ability to repair and rebalance via optimal gene expression [7]. This focus on medicating abstinence seems counterintuitive. Health can only be nourished, not forced; this is more easily said than done. In this multi-billion-dollar market, Big Pharma opted for simplicity, combating the global drug epidemic by blocking dopamine function with drugs like naltrexone (via mu receptor antagonism) or with, for example, Acamprosate, via antagonizing the NMDA–glutaminergic drive to release dopamine at the reward site (nucleus accumbens) [8].

Mark Gold and associates in their “dopamine depletion hypothesis” [9] suggested that the powerful dopamine two receptor (DRD2) agonist bromocriptine could be used for the treatment of cocaine addiction [10]. Fortunately, neuroscientists realized that chronic administration of powerful D2 agonists caused a severe reduction in the number dopamine receptors (down-regulation) [11]. The reason for this unwanted side effect is that bromocriptine or other powerful D2 agonists like L-Dopa overwhelm the neural pathways of the brain, especially the pleasure centers, and the biologically intelligent neurochemical adaptive mechanisms react to prevent too much dopamine function (hyperdopaminergia) and, possibly, schizophrenic–like behaviors [12].

Blum and associates have been developing, by trial and error and genetic testing, a neuro adaptogen, KB220, and many subsequent improved variants, since 1968 [13]. To date, 37 published clinical studies have validated this nutrient technology based on gene mapping research. This patented technology is comprised of a list of ingredients intended to optimize gene expression and the synthesis, transport, reception, and disposal of neurotransmitters [14]. Optimization of gene expression for each neurotransmitter in the entire brain reward cascade, from serotonin in the brain stem to dopamine release in the nucleus accumbens/basal ganglia and cortical regions, achieving the functional ‘symphony of neurochemistry’ and the induction of “dopamine homeostasis” [15].

The first ever confirmed psychiatric genetic discovery by the Blum and Noble’s group, the association of the Dopamine D2 Receptor (DRD2) gene and severe alcoholism, was published in JAMA in 1990 [16]. The association in genetic studies of the DRD2 gene with many addictions, such as, alcohol, drugs, food, sex, nicotine, and other excessive reward seeking or self-medicating behaviors led to the idea of “Reward Deficiency Syndrome” (RDS), first coined by Kenneth Blum in 1995 [17]. Reward Deficiency Syndrome is now considered to be an established abnormal psychological syndrome listed in the SAGE Encyclopedia of Abnormal and Clinical Psychology (2017) [18] and refers to a deficiency of reward, paired with disrupted neurological dopamine function. This dysregulation of dopamine is the proposed cause of most (and, perhaps, all) all addictive, compulsive and impulsive behaviors.

**Figure 2:**
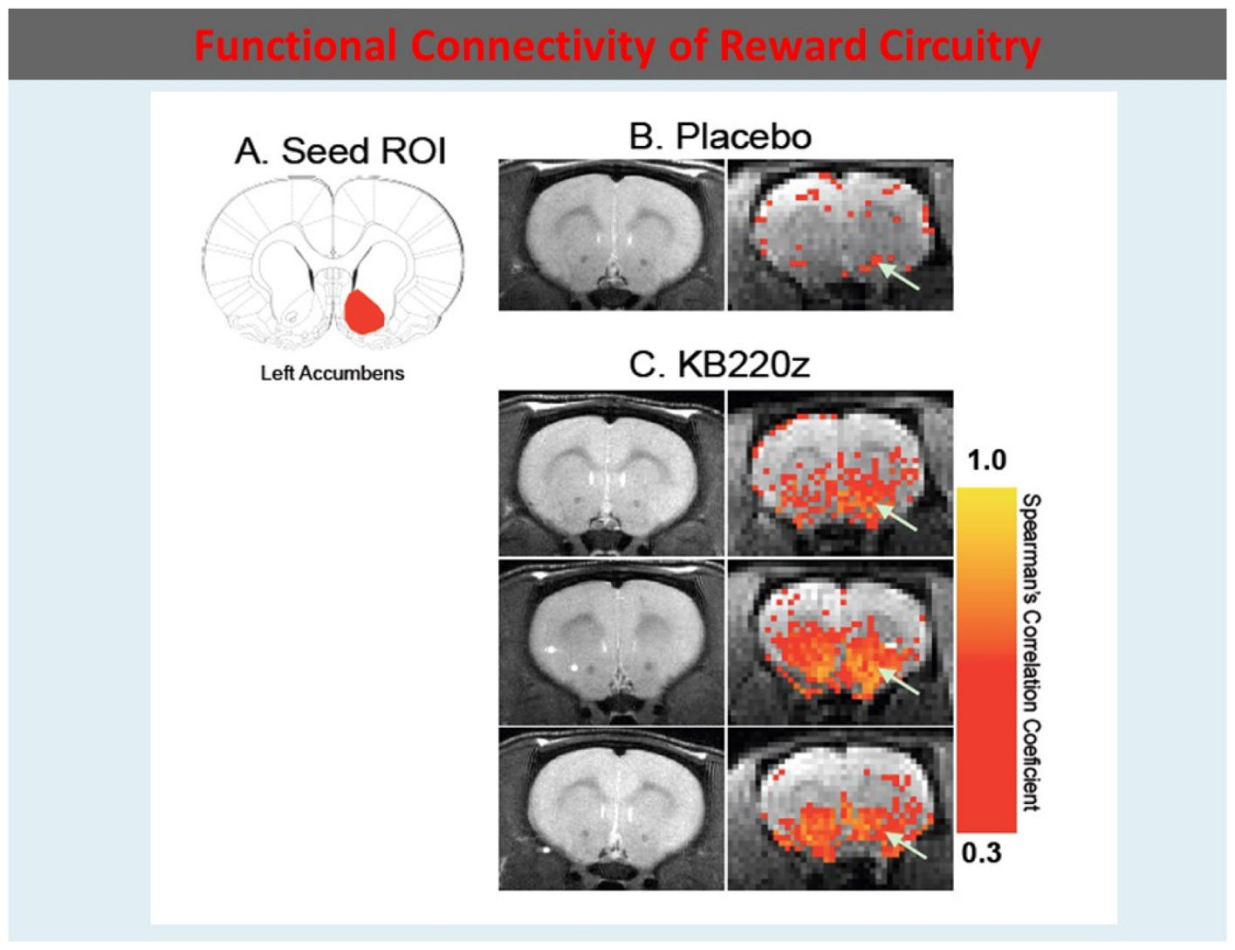
In rats KB220Z compared to Placebo seed Region of interest (ROI) os the left Accumbens

To highlight the importance of the RDS concept, in 2013, B. William Downs and Kenneth Blum, published a paper entitled “*Have We Hatched the Addiction Egg: Reward Deficiency Syndrome Solution System?” This paper was* dedicated to all the people who have lost loved ones to substance abuse and “reward deficiency syndrome”-related tragedies. Why are we failing at reducing the incidence of RDS behaviors? Are we aiming at the wrong treatment targets? At that time, a paradigm shift was proposed; the *“Reward Deficiency Solution System”* and evidence was provided for its adoption. The “*Reward Deficiency Syndrome Solution System”* provided evidence for the feasibility of its adoption [19,20]. While the RDSQ and GARS are in development and should be launched in 2018, the patented foundational ingredients of the KB220/Z/ZBR formulas have been studied in both animal (see Figure 2) [21] and human research (see Figure 3) [22]. After 50 years of study of brain reward systems, we now have fMRI evidence that KB220/Z/ZBR variants can enhance resting state, functional connectivity and brain connectivity volume (recruit neuronal firing in the reward center of the brain) [21] and balance the brain reward circuitry, especially, in abstinent heroin-dependent people.

Unfortunately, a number of companies have decided to include L-Dopa as an ingredient in their nutraceutical to affect craving behavior. While the product contains some of the same ingredients as found in KB220 variants, the inclusion of the drug L-Dopa, an amino acid precursor of dopamine approved by FDA for Parkinsonism, with well documented side effects, is of concern. Of even more concern is the potential of the tainted product to cause more harmful effects, common to products identified as “dietary supplements”. A possible harmful effect notwithstanding, this ingredient disrupts the neurochemical balance, especially of dopamine, and can induce unwanted hyperdopaminergia and dyskinesia, instead of muchneeded balance [23]. It has the same effects as bromocriptine leading to dopamine D2 receptor down-regulation [24]. There is evidence that chronic administration of L-Dopa increases prefrontal cortex dopamine and serum corticosterone (a stress-related hormone) [25] and could enhance aberrant signaling in the D1 pathway [24] in denervation states, which arguably could be the case in the presence of chronic antagonists or reward deficiency. There is also evidence of a profound serotonin-dopamine imbalance, following L-Dopa treatment [26]. Some manufacturers, despite FDA restrictions, have produced plant-based L-Dopa in the form of Velvet Bean (Mucuna pruriens) and studies have linked this to both psychosis and homicidal behavior [27,28].

Over 100 million people in the United States carry the D2 receptor gene A1 allele [29], which is responsible for lower D2 receptor formation, is present in Parkinson’s disease and may be a precursor to the development of drug-induced dyskinesia, and potentially confer risk for Alzheimer’s disease [Blum et al., 2017]. Therefore, persons presenting for chemical dependency treatment should be warned about using any product containing L-Dopa. Moreover, low dopamine function can be problematic, specially, in carriers of the valine allele (replacement of methionine) that causes reduced dopamine function due to the high activity of synaptic dopamine break down [30]. This high activity could subsequently produce an unwanted potent neurotoxin metabolite from L-Dopa in the form of 3-O-methyldopa [31]. Another associated problem with L-Dopa administration is that it is known to cause a decrease in concentrations of S-Adenosyl methionine (SAM-e) [32] in cerebrospinal fluid with an increase in 3-methoxytyrosine, especially, in children. The small molecule, SAM-e, is involved in a process known as methyl donation, seen as an intermediate in one pathway to epigenetic cellular maintenance.

Known side effects of the chronic administration of L-Dopa for Parkinson’s patients include mania, dyskinesia (rigidity in extremities, face, mouth, and tongue), and abnormal involuntary movements (AIMs). Also, reported among the side effects are psychosis, auditory hallucinations, homicidality, hypersexuality, confusion, delusions, orthostatic hypotension, sleep disruption, age-related mental disturbances/cognitive decline, impaired gait, and kaliuresis (renal dysfunction with the induction of unwanted excretion of potassium) are side effects Based on ignoring many studies, the use of L-Dopa is still considered Generally Recognized as Safe (GRAS), but the FDA has provided limitations on the over-the-counter use of L-Dopa and even the associated plant extracts [33]. Certain combinations that evoke significant caution include: threonine, a GRAS listed amino acid precursor, in combination with L-Dopa, are present in products that claim benefit for anti-cravings. L-theanine increases neurotransmitter production, one of which is dopamine. L-theanine (N-ethyl-L-glutamine) or theanine is an amino acid found in green teas. Historically, L-theanine has been reported to be a relaxation-promoting agent. This has prompted some scientific research on its underlying pharmacology. Animal neurochemistry studies suggest that L-theanine increases brain serotonin, dopamine, GABA levels and has micromolar affinities for AMPA, Kainate, and NMDA receptors [34]. Green tea has lots of threonines, although it can also be taken as a supplement. Along these lines, Acetyl-l-tyrosine (a potential supplement) is a production-ready form of tyrosine promoting brain dopamine synthesis. It is easy to understand that this combination of threonine and L-Dopa is unwanted, especially, in any nutraceutical supplement with the potential to impact the over-production of dopamine or even GABA, within the central nervous system and peripherally.

## Summary

The well-researched, original pro-dopamine regulator, KB220, and variants (i.e. KB220Z/ZBR) utilizing recent technical advancements, show increased functional connectivity, in both animal and human neuroimaging studies. Prolonged neuroplasticity (neurogenesis) has been observed in rodents. Moreover, studies have been published showing that KB220Z increased function and brain connectivity volume, enhanced neuronal dopamine firing, and has eliminated lucid nightmares in humans over a prolonged period. An unprecedented number of clinical studies validating the success of a patented nutrigenomic technology to re-balance brain chemistry and optimize dopamine sensitivity and function have been published. The patented formula is the culmination of decades of meticulous trial and error tests on the effects of ingredients individually and on a plethora of combinations in animals and humans to determine the best behavioral benefits and outcomes. Subsequently, the formula outcomes were honed and verified by evaluating improvements in gene expression. Other ingredients have been included to expand the mechanistic reach to optimize gene expression for improved functionality of the neuro-endocrine-immune axis; optimizing the harmonious connectivity between different brain regions to achieve the symphony of neurochemistry. The improved effectiveness of the formulas has been further validated in clinical research including numerous brain scan studies published in peer-reviewed and cited scientific journals. Adding pro-hormones, plant-based hormonal analogs, or bio-identical hormonal substances to the exhaustively and meticulously researched and developed KB220Z/ZBR formulation will only disrupt the elaborate and sequential flow of genetic communication sequelae and the hamonious interconnectivity between neurotransmitters and their homeostatic, regulatory feedback controls in the Brain Reward Cascade. Due to these feed back regulatory controls, such additions result in homeostatic downregulation of neurotransmitter synthesis and reception to compensate for the artificial presence of such pseudoneurotransmitter substances. It is a similar reactive mechanism, when prednisone is introduced to the system, the body stops making cortisone. The same mechanism applies to insulin, thyroid, and any neurotransmitter, especially, dopamine.

The KB220Z/ZBR nutrigenomic formulas (and prior variants) have been formulated to re-balance, harmonize, and optimize neurotransmitter functional relationships in the Brain Reward Cascade-not displace, replace, or disrupt those functional relationships. Moreover, products containing ingredients having potential dangers (i.e., combinations of potent D2 agonists, including L-Dopa and L-Theanine) threaten the credibility and reputation of validated, authentic, and ethical products and may cause unwanted harm to the unsuspecting subject. We encourage clinicians and neuroscientists to continue to embrace the concept of “dopamine homeostasis” and search for safe, effective, validated and authentic means to achieve a lifetime of recovery, instead of reverting to anti-dopaminergic agents doomed to fail in the war against this devastating drug epidemic.

## Figures and Tables

**Figure 3:**